# Genotype-by-Environment Interactions for Female Mate Choice of Male Cuticular Hydrocarbons in *Drosophila simulans*

**DOI:** 10.1371/journal.pone.0067623

**Published:** 2013-06-25

**Authors:** Fiona C. Ingleby, John Hunt, David J. Hosken

**Affiliations:** 1 Centre for Ecology and Conservation, School of Biosciences, University of Exeter, Cornwall Campus, Tremough, Penryn, United Kingdom; 2 University of Sussex, John Maynard Smith Building, Falmer, Brighton, United Kingdom; University of Freiburg, Germany

## Abstract

Recent research has highlighted the potential importance of environmental and genotype-by-environment (G×E) variation in sexual selection, but most studies have focussed on the expression of male sexual traits. Consequently, our understanding of genetic variation for plasticity in female mate choice is extremely poor. In this study we examine the genetics of female mate choice in *Drosophila simulans* using isolines reared across two post-eclosion temperatures. There was evidence for G×Es in female choosiness and preference, which suggests that the evolution of female mate choice behaviour could differ across environments. However, the ranked order of preferred males was consistent across females and environments, so the same males are favoured by mate choice in spite of G×Es. Our study highlights the importance of taking cross-environment perspectives in order to gain a more comprehensive understanding of the operation of sexual selection.

## Introduction

Female mate choice exerts strong sexual selection on males and is thought to drive the evolution of many elaborate sexual traits and displays [1]. Despite an initial reluctance to recognise the importance of mate choice in sexual selection [2], [3], research in this area has advanced and female mate choice has been documented in many species and is understood in considerable detail [1], [4]. Studies have demonstrated that females can benefit from mate choice directly through resources provided by the male [5], or indirectly via offspring gaining viability or attractiveness genes [6], [7], [8], [9]. However, given the evidence for plasticity and context-dependency of mate choice in a wide range of species [1], [10], it seems unlikely that mate preferences will be static and that all females will prefer the same males in every environment.

Unfortunately little is known about the genetics underlying plasticity in mate choice, and we therefore have a very limited understanding of the operation and evolution of mate choice across heterogeneous environments. The potential significance of this plasticity in mate choice has been highlighted by recent interest in genotype-by-environment interactions (G×Es) in sexual selection [11], [12]. G×Es describe changes in the relative performance of genotypes across environments [13], and have been studied within the field of evolutionary genetics for well over twenty years [14]. Interest in G×Es in a sexual selection context is more recent, but theoretical and empirical work suggests that G×Es could be of fundamental importance to the operation of sexual selection [12].

Theory suggests that G×Es in the expression of male sexual signals and displays can make sexual signals unreliable [15], but can also contribute towards the maintenance of genetic variation in sexual traits [16], and there is some empirical support for these predictions [17], [18], [19], [20]. Far fewer studies have considered G×Es in female mate choice, although G×Es in mate choice could also have important implications for sexual selection. A G×E for mate choice demonstrates that there is genetic variation for plasticity in mate choice across environments, and thus the evolution of mate choice will vary across environments. In this way, G×Es could enable adaptive plasticity in mating decisions [16]. For example, G×Es in male sexual signals might not result in signal unreliability across heterogeneous environments if there are also G×Es in female mate choice. If the reaction norms for female mate choice and male signals match one another, then changes in the direction and extent of signal plasticity will be mirrored by changes in mate choice across environments, and benefits of mate choice could therefore be maintained in spite of G×Es for trait and preference [11]. On the other hand, if reaction norms for female mate choice and male signals do not match, then signal reliability could be disrupted because the genetic covariance between signal and preference could vary in strength and sign between environments. Sexual selection by a Fisherian runaway process is to a large extent determined by the strength of genetic correlation between female preference and male sexual trait [3], [6], and so environmental heterogeneity and G×Es in preferences and signals could affect the operation of sexual selection.

Empirical studies suggest that G×Es in male sexual traits are widespread [e.g. 21,22,23,24], although not ubiquitous [e.g. 25,26]. However, very few studies have examined G×Es in female mate choice. Those that have find evidence for G×Es in aspects of mate choice behaviour in the waxmoth, *Achroia grisella* [27], and in *Drosophila melanogaster* [28], but there is no evidence for G×E in *D. serrata* mate preferences [29]. Further study is therefore needed to determine how common G×Es for female mate choice are, and explore their consequences for sexual selection.

Here, we test for G×Es and examine the genetics of female mate choice in *D. simulans*. Previous work has shown that there are no direct costs or benefits of mate choice in this species [30], but that females benefit indirectly through heritable male attractiveness [9], [31]. Furthermore, we have evidence that male attractiveness is heritable across environments, although aspects of male sexual signalling with cuticular hydrocarbons (CHCs) are unreliable [20] because of G×Es in male CHC expression [19], [20]. Based on the results of these previous studies of mate choice in *D. simulans*, we expect that there will be genetic variation for female mate choice and that mating decisions will be influenced by male CHC profile. Further, evidence that CHC expression is strongly influenced by temperature variation in our population of *D. simulans* [19], [20] makes it likely that mate choice behaviours will also be influenced by temperature. This study provides an in-depth analysis of variation in female mate choice across genotypes and environments.

Using females from isolines reared across two temperature environments, we examine two important aspects of female mate choice: choosiness and preference. We use these terms exactly as defined by Jennions and Petrie [1] and consistent with Cotton et al. [10]. Preference describes the willingness of a female to mate, which we interpret here from mate acceptance data (a binary measure of whether or not a female mates within a given period of time). Choosiness describes the time and effort a female spends assessing potential mates [1], [10], and we interpret this from copulation latency, which is the time between introduction of a male and female and the start of mating, and is a common metric used in *Drosophila* studies [e.g. 9,28,31,32,33,34].

We assay mating behaviour in trials with single males and females (i.e. the choice is whether or not to mate with a given male; *sensu* Shackleton et al. [35]) since this allows us to uncouple mate choice from male-male competition, which would be confounded in trials using multiple males. Note also that in *Drosophila*, studies using single and multi-male assays produce identical results [e.g. 30,36]. This study therefore quantifies genetic variation and plasticity in two aspects of female mate choice behaviour across two temperature environments. Additionally, male CHC expression is measured, which allows us to analyse female mate choice as a function of a male sexual signal, as well as test the genetic covariance between female preference and male attractiveness across environments.

## Materials and Methods

### Isolines and Maintenance

Female *D. simulans* were collected from Greece in April 2010 and used to found 60 iso-female lines (isolines) in the laboratory. No specific permits were required for this work as *D. simulans* is a cosmopolitan human commensal that is distributed world-wide. Within each isoline, 25 male and 25 female offspring were used to found each new generation. Isolines had been maintained in this way for 34 generations prior to this experiment and so each isoline can be considered a distinct genotype to some extent. Isolines were maintained on a standard cornmeal-based diet (supplied by Applied Scientific, UK) at 25°C on a 12∶12 hour light:dark cycle throughout the experiment (unless stated otherwise).

We used a subset of 28 isolines in this study, 8 of which were used to derive experimental males (henceforth referred to as ‘male isolines’). These male isolines were chosen based on results from previous experiments [19], [20], in order to provide male genotypes with broad variation in attractiveness. The other 20 isolines were used to derive experimental females (‘female isolines’) and had been chosen haphazardly from the remaining isoline stock, such that males and females were derived from different isolines.

### Environmental Manipulation and Mating Assays

The experiment was carried out in 7 blocks. For each block, adult flies were taken from each of the male and female isolines and used to set up two replicate laying vials per isoline, each with five males and five females in 150 ml vials with 30 ml of food. After a 48-hour laying period, the adult flies were removed and the vials were incubated at 25°C during offspring development. Development took 11 days until peak eclosion, at which point virgin flies were collected. Any eclosed adults were cleared from vials at 7 am. Newly-eclosed virgin adults were collected between 11 am and 1 pm, and again between 5 pm and 7 pm. Virgin males were collected from each of the 8 male isolines and housed by isoline (10 males per 40 ml vial with 8 ml of food) at 24°C, to create tester males from a standard environment which was distinct from the experimental environments, to avoid any effect of common environment between males and females. All males were kept at the same temperature and same density to minimise male variation due to environmental factors. From each of the 20 female isolines, virgin females were collected and housed individually in a 40 ml vial with 8 ml of food. Females were split approximately equally between two post-eclosion temperatures, 23°C and 25°C. These temperatures were chosen to represent an unstressful range which flies would frequently experience both in the lab and in their natural environment. This narrow range of temperature is therefore both biologically relevant, as well as representing a difference in temperature which is known to have a significant effect on *D. simulans* CHC expression [19], [20].

These males and females were used in mating assays which were carried out at 3 days post-eclosion. Over the entire experiment, 6–8 females from each female isoline×environment combination were assayed with a male from each of the male isolines (6–8 replicate females from 20 female isolines×2 post-eclosion temperatures×8 male isolines = 2239 assays, carried out in 7 approximately equal blocks). Each assay was carried out at 24°C and lasted 3 hours, during which courtship and mating behaviour was observed. Pairs not observed attempting courtship were excluded from the analysis. For the pairs that did court, we recorded whether or not they successfully mated, and the time when they started to mate. This provided mate acceptance data (as a binary measure of whether or not a pair successfully mated during the 3-hour assay) and copulation latency data (the time between introduction and the start of copulation). Copulation latency measured this way is highly positively correlated with latency between first courtship and copulation [9], but is easier to accurately observe and record. In *Drosophila*, females have control over acceptance or rejection of courting males [34], [37], [38], and so preferred males should copulate more rapidly. From our data, we therefore had two measures of overall mate choice for females from each female isoline×male isoline×environment combination. Consistent with definitions given by Jennions and Petrie [1], we interpret variation in copulation latency as variation in female choosiness, and variation in mate acceptance as variation in female preference.

### Assessing Male CHC Profile

CHCs are waxy compounds produced on the adult cuticle of many insects and have been shown to be important sexual signals in many *Drosophila* species [39]. Two sets of virgin males for CHC profiling were also collected during virgin collection (see above). Firstly, males were collected from each of the 8 male isolines (56–63 males from each isoline, *N* = 485) to provide CHC data for the male genotypes used in the mating assays. This allowed us to examine female preference for these genotypes as a function of average male CHC profile. We did not sample CHCs from the same individuals used in the mating assays since CHC profiles can change with mating [39], [40]. However, the CHCs sampled from virgin males from the same isolines will closely represent the CHC profiles of virgin males used in the assays since the isolines are very heavily inbred, and we reared males for mating assays and CHC profiling in identical environmental conditions (10 males per 40 ml vial with 8 ml of food kept at 24°C), and both mating behaviour and CHCs were assayed at 3 days post-eclosion. The second set of males were collected from each of the 20 female isolines (12–14 males per isoline, *N* = 270). These males were split between the same two post-eclosion temperatures as the females from these isolines (23°C and 25°C). Males were housed together according to isoline and temperature in 40 ml vials with 8 ml of food. CHC profiling of these males gave us male CHC data from each female isoline×environment combination, and, in combination with the data on female mate choice from the same female isoline×environment combinations, allowed us to calculate the genetic covariance between male sexual signal and female mate choice for these 20 isolines across both temperatures.

Males for CHC profiling were transferred to individual glass auto-sampler vials (supplied by Chromacol, UK) at 3 days post-eclosion, and stored at −80°C prior to hydrocarbon extraction. Hydrocarbon extractions were carried out in sets of 100 samples per day, and randomised throughout by isoline and environment. Hydrocarbon extractions and analysis followed a protocol optimised previously for *D. simulans* [19], [20]. Hydrocarbon extraction involved soaking each fly in 50µl of a solution of 10 ppm penta-decane in HPLC-grade hexane for 5 minutes. Penta-decane was added as an internal standard. A vortex was used for the duration of the final minute to agitate to solution and maximise the extraction. The fly was then removed from the vial using forceps sterilised in hexane. 2µl of each hydrocarbon sample was injected into a GC-FID (Agilent 7890) fitted with two injectors, and two DB-1 columns of 30 m×0.25 mm internal diameter×0.25 µm film thickness. We used hydrogen as a carrier gas. The inlet was set at 250°C, and the injection was in pulsed splitless mode. Separation of the extract was optimised using a column profile, which began at 70°C for 1 minute and then increased at 20°C per minute to 180°C, then 4°C per minute to 220°C and finally 15°C per minute to 320°C, where it was held for 2 minutes. The FID detector heaters were set at 300°C. The hydrogen flow was 20 ml per minute, and the air flow was 200 ml per minute. Nitrogen was used to make up the column flow to 30 ml per minute. Peak integration of the hydrocarbon data was carried out using GC ChemStation software (version B.04.02.SP1).

### Statistical Analyses

All analyses were carried out using R (v.2.13.0) and copulation latency (female choosiness) and mate acceptance (female preference) were analysed separately. Mate acceptance was scored as 0 (unmated) or 1 (mated) (*N* = 2239), and copulation latency (seconds elapsed between introducing the male to the vial and the start of copulation) was log-transformed prior to analysis to fit a normal distribution. Copulation latency was analysed using only the pairs that successfully mated during the assay (*N* = 1674).

### Model Fit and Evaluation

We used generalised linear mixed models and Bayesian inference as implemented by the MCMCglmm package v.2.12 [42]. Temperature was specified as a fixed effect, and female and male isoline as random effects. We used a Gaussian distribution for the copulation latency data and a ‘categorical’ distribution (in MCMCglmm notation) to handle the binary mate acceptance data. For each model, we ran Markov chains for 400,000 iterations with a burn-in of 20,000 and a thinning interval of 25. Each model used unstructured variances (‘us’ in MCMCglmm notation), therefore estimating all variance and covariance parameters. We tested models both with an informative (*ν* = 2) and a relatively uninformative prior (*ν* = 0.02) and found that results were robust to changes in prior specification. We present results from models with relatively uninformative priors (*ν* = 0.02), which means that models were fitted with very little *a priori* information about the expected parameter estimates.

A set of 7 plausible models were tested for each response, which examined combinations of male isoline, female isoline, environmental and G×E components of mate choice (see Table 1 for the biological rationale of each model). Statistical support for each model was estimated using the deviance information criteria (DIC), and also by calculating the approximate posterior probability. This calculation takes into account the DIC of each model tested, and for each, provides a probability that can be used to identify the best approximating model out of the set being tested. Models were tested with and without experimental block as a covariate, but inclusion of a block term did not alter our results and model fit was consistently better without a block term (Table 1), and so further analyses do not include block.

**Table 1:** Summary of the sets of models tested for (I) female choosiness (copulation latency) and (II) female preference (mate acceptance) data.

	Model rationale	Variancestructure	DIC (posterior probability) without block	DIC (posterior probability) with block
**I. ** ***Female choosiness***				
**1.**	**Genetic variation for both choosiness and attractiveness**	**F+M**	**4153.165 (0.852)**	**4155.402 (0.839)**
2.	G×E for female choosiness and G for male attractiveness	F×t+M	4156.730 (0.143)	4158.780 (0.155)
3.	Female genetic and environmental variation for ranked order of male isolines	F×M+M×t	4163.460 (<0.001)	4166.307 (<0.001)
4.	Genetic variation for male attractiveness	M	4172.226 (<0.001)	4174.435 (<0.001)
5.	Female genetic variation for ranked order of male isolines	F×M	4195.469 (<0.001)	4196.404 (<0.001)
6.	Female G×E for ranked order of male isolines	F×M×t	4198.091 (<0.001)	4200.514 (<0.001)
7.	35. Genetic variation for female choosiness	F	4257.801 (<0.001)	4260.257 (<0.001)
**II. ** ***Female preference***				
**1.**	**G**×**E for female preference and G for male attractiveness**	**F**×**t+M**	**2363.753 (0.986)**	**2365.790 (0.963)**
2.	Genetic variation for both preference and attractiveness	F+M	2372.393 (0.013)	2374.284 (0.037)
3.	Female genetic and environmental variation for ranked order of male isolines	F×M+M×t	2395.824 (<0.001)	2398.063 (<0.001)
4.	Female genetic variation for ranked order of male isolines	F×M	2406.907 (<0.001)	2409.905 (<0.001)
5.	Female G×E for ranked order of male isolines	F×M×t	2408.196 (<0.001)	2409.950 (<0.001)
6.	Genetic variation for female preference	F	2441.910 (<0.001)	2443.997 (<0.001)
7.	Genetic variation for male attractiveness	M	2449.585 (<0.001)	2450.941 (<0.001)

All models include post-eclosion temperature (t) as a fixed effect. Female isoline (F), male isoline (M) and any interactions are added as random effects, as shown. The best model is highlighted in bold and chosen using the DIC (supported by the approximate posterior probability) and models are ranked from best model fit (lowest DIC) to poorest model fit (highest DIC). Results are shown for models with and without block as a covariate. Results are qualitatively identical with and without a block effect, but model fit is improved slightly by removing block.

### Model Interpretation

Reaction norms were plotted to illustrate female isoline×temperature interactions for both copulation latency (female choosiness G×E) and mate acceptance (female preference G×E). For latency and acceptance individually, we estimated the cross-environment genetic correlation, heritability between and within environments, and variance components for female isoline, male isoline and female isoline×temperature. These estimates were made from the simplest model to include all the relevant parameters (i.e. female isoline×temperature+male isoline; see Table 1). Genetic correlation, heritability and variance components were calculated following Lynch and Walsh [13]. By using Bayesian inference, we were able to extract 95% credible intervals around each of these estimates (directly from the posterior distribution of the models). These estimates therefore account for uncertainty in the data and allow us to test if each estimate is significantly different from 0 or 1 (ie. whether or not the credible interval overlaps with 0 or 1).

The male isoline term in the models in Table 1 was interpreted as genetic variation in male attractiveness. Both measures of female mate choice can vary without necessarily affecting the order in which male isolines are ranked, and so to test variation in how males were ranked, we included interactions in the models. An interaction between female rearing temperature and male isoline (M×t in Table 1) would suggest that the ranking of male genotypes varied depending on female rearing environment, whereas an interaction between female isoline and male isoline (F×M) would suggest (female) genetic variation for how male genotypes were ranked. A three-way interaction between female isoline, female rearing environment and male isoline (F×t×M) would suggest G×E variation for the ranking of males.

The CHC data for the male isolines was used to examine mate choice in terms of male CHC phenotype (as opposed to male genotype as above). Expression of 22 hydrocarbon peaks was quantified for each male. We calculated relative peak size by dividing each peak by an internal standard (pentadecane) within each sample, and then normalised the CHC data using log transformation prior to analysis, creating log contrasts for each CHC. The pooled male CHC data from both male and female isolines was used in a principal components analysis (PCA) to reduce the dimensionality of the data and extract the same vectors of CHC variation for males from male and female isolines. PCs were extracted from the correlational matrix and vectors with eigenvalues greater than 1 were used in subsequent analyses. This gave four PCs which together explained 83% of the total variation in CHC expression. We plotted copulation latency (choosiness) and mate acceptance (preference) for each female isoline as a function of male CHC profile (using the ranked PC scores for each male isoline) with the ‘smooth.spline’ function in R (‘stats’ package).

The set of models in Table 1 was re-analysed without the male isoline term, instead using the four PCs of male CHC profile as covariates to account for male effects on female mate choice in terms of male phenotype. Since our measure of CHC expression is an average CHC profile for each male isoline, the best models for both copulation latency and mate acceptance using male CHC data are analogous to the best models identified using the male isoline term. From the posterior distribution of the best model for each response, we were able to estimate overall *β*, the linear selection gradient, to quantify sexual selection through mate choice on each PC. In addition, an estimate of *β* (for each PC) for each female isoline×temperature combination was also extracted from the posterior distribution of the model including the female isoline×temperature×male isoline interaction, in order to examine genetic and environmental variation in *β*.

### Genetic Covariance between Female Preference and Male Attractiveness

The cross-environment genetic covariance between female preference and male attractiveness (calculated from CHC profile) was analysed using the male and female data from the 20 female isolines in each post-eclosion temperature. For males from each female isoline×environment combination, a mean attractiveness score was assigned based on CHC profile. These attractiveness scores were calculated from the results of a discriminant function analysis of PCs 1–4 of CHC expression for the males from the male isolines that were used in the mating assays, using mate acceptance (0 or 1) as the response (using the ‘lda’ function in the ‘MASS’ package in R). The discriminant function identified the vector of male CHC variation that best distinguished between mated and unmated males and could therefore be used as a surrogate of the attractiveness of a male's CHC profile. Since both sets of male CHC data were pooled for PCA (see above), we had characterised the same 4 PCs for males from the female isolines as we did for the males from the male isolines. The data for the males from the female isolines could therefore be directly projected onto the vector identified by the discriminant function analysis, providing a univariate attractiveness score for males from each female isoline×environment combination.

Using the MCMCglmm package as before, we tested for G×E in male attractiveness scores across temperatures. Models were specified as described above. We used a Gaussian distribution, with temperature as a fixed effect and female isoline as a random effect. We tested two models: one including a G×E (female isoline×temperature) for male attractiveness score, and the other with only G and E effects. Using the same methods described above, we assessed model fit, and calculated the cross-environment genetic correlation of male attractiveness score from the model that included the G×E term.

The genetic correlation between female preference and male CHC attractiveness both within and across temperatures was calculated following Lynch and Walsh [13], using mean female mate acceptance and mean male CHC attractiveness scores for each female isoline×temperature combination. Genetic correlations were calculated with bootstrapped 95% confidence intervals and significance assigned by randomisation test (with 10,000 iterations).

## Results

### Female Choosiness

Consistent with previous definitions of female mate choice [1], [10], we interpret variation in female choosiness using the copulation latency data.

**Figure 1:**
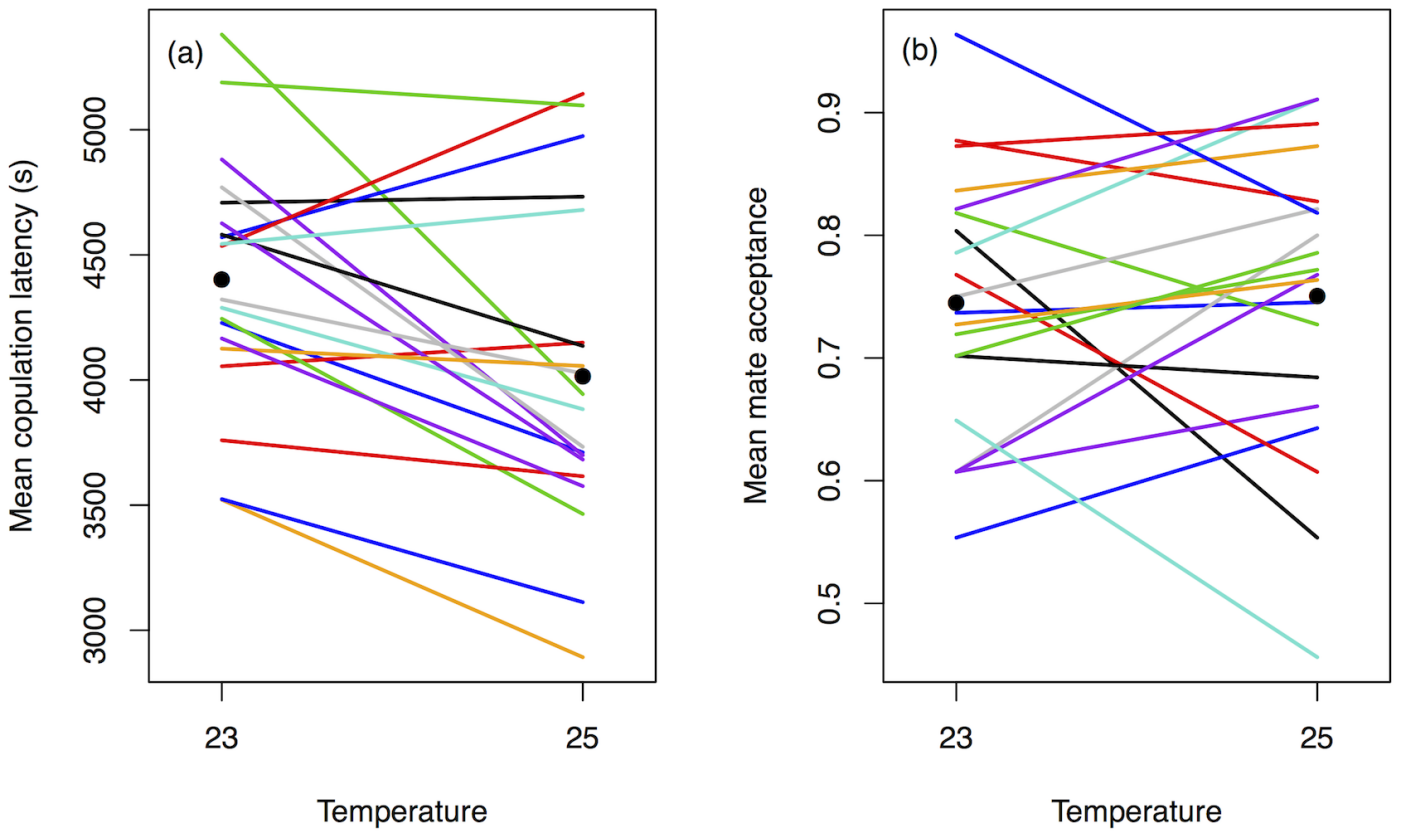
Female G×E reaction norms for *(a)* female choosiness (copulation latency); and *(b)* female preference (mate acceptance) across post-eclosion temperatures. Each coloured line represents the mean score for each female isoline (*N* = 20 isolines). Points represent the overall mean score within each temperature across all isolines.

**Figure 2:**
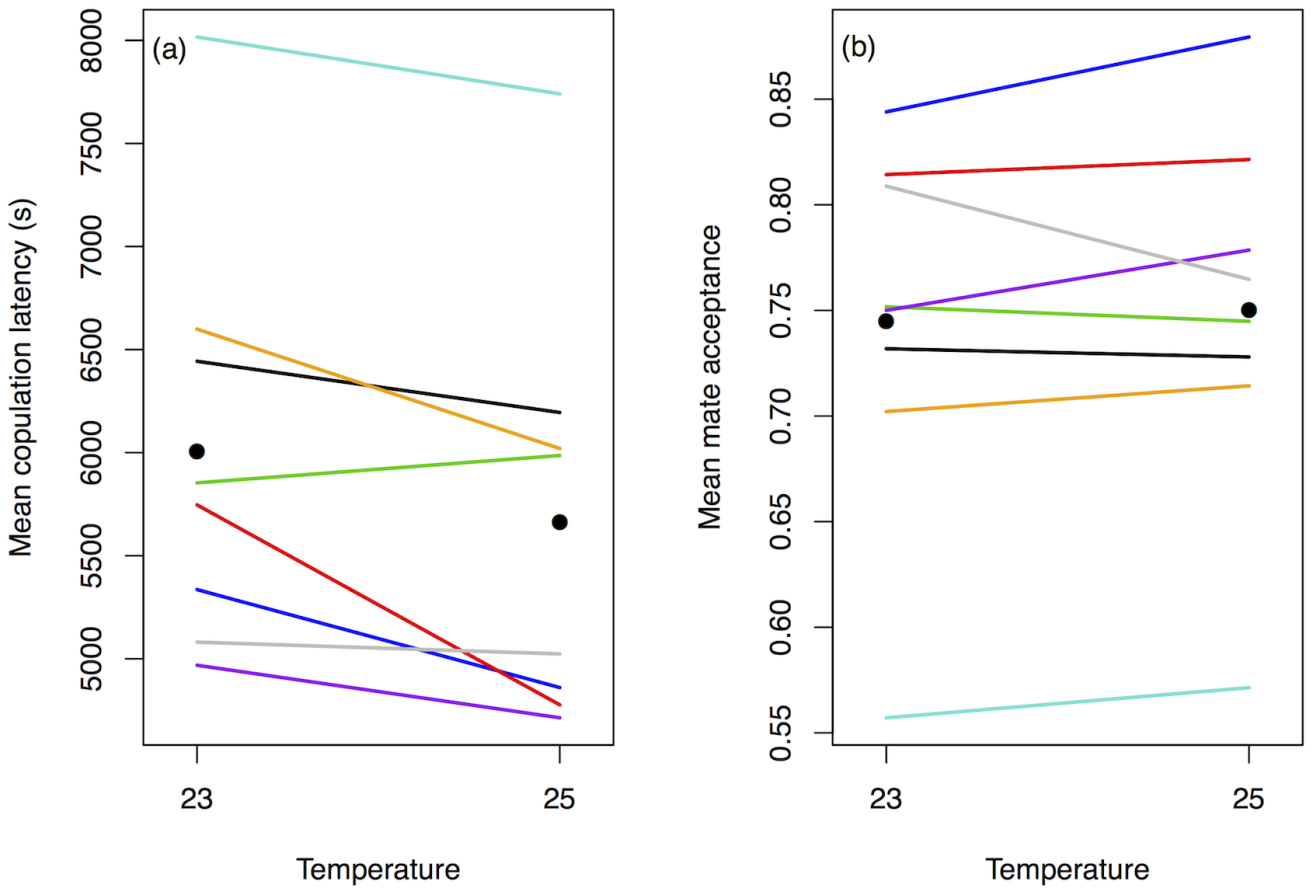
Male isoline attractiveness ranked by (a) female choosiness (copulation latency); and (b) female preference (mate acceptance) across female post-eclosion temperature. Each coloured line represents the mean score for each male isoline (*N* = 8 isolines). Points represent the overall mean score within each temperature across all isolines. Note that the interaction between male isoline attractivness and female rearing temperature (M×t) was not included in the best model for either female choosiness or female preference.

The model with the strongest support (85.2%) for this data (Table 1) shows genetic variation in both female choosiness (‘F’ term in Table 1) and male attractiveness (‘M’). Genetic variance in female choosiness is low, while genetic variance in male attractiveness is high (Table 2). Females reared at 25°C mate more quickly than females reared at 23°C (Figure 1a) although the credible intervals around these estimates overlap (fixed effect estimate [with 95% credible interval] for 25°C: 7.93 [7.70–8.11]; and for 23°C: 8.20 [7.98–8.41]). Despite variation in female choosiness and male attractiveness, this model shows that the ranked order of males remains the same across all female genotypes and environments (since there were no F×M, M×t or F×M×t interactions in the best model; see Figure 2a), but that females mate more readily in the warmer environment. The lack of G×E for female choosiness (female isoline×temperature) in the best model suggests that the effect of temperature on female choosiness does not vary significantly between female genotypes. This is reflected in the low variance explained by the G×E interaction (Table 2). However, despite the lack of significant G×E, crossover can be seen in the reaction norms in Figure 1a, and there is evidence for substantial changes in genetic variation in choosiness between temperatures (Table 3). In fact, both the cross-environment genetic correlation and the between-environment heritability are significantly lower than 1 but not significantly different from 0, showing a very weak genetic correlation across environments and very low heritability between temperatures, although the intervals around these estimates are wide. Note also that the model that includes a G×E for choosiness had some statistical support (14.3%) and the difference in DIC between the G×E model and the best model for choosiness (which excluded G×E) was small.

**Table 2:** Variance in female choosiness (copulation latency) and female preference (mate acceptance) accounted for by male isoline, female isoline and female isoline×temperature (G×E).

	*Choosiness*	*Preference*
Male isoline	**0.084 (0.024–0.258)**	**0.421 (0.111–1.299)**
Female isoline	**0.017 (0.005–0.040)**	**0.425 (0.176–0.902)**
Female isoline×temperature	0.004 (0.001–0.013)	**0.090 (0.008–0.256)**

95% credible intervals around each estimate are in brackets. Components included in the best model for each response are highlighted in bold.

**Table 3:** Cross-environment genetic correlation and between- and within-environment heritability of female choosiness (copulation latency) and female preference (mate acceptance).

	*Choosiness*	*Preference*
Genetic correlation, *r_g_*	**0.606 (**−**0.115–0.934)**	**0.679 (0.130–0.982)**
Heritability, *H^2^*:		
within 23°C	0.752 (0.209–1.435)	0.850 (0.368–1.390)
between temperatures	**0.555 (**−**0.097–0.906)**	**0.646 (0.123–0.961)**
within 25°C	1.248 (0.565–1.791)	1.150 (0.610–1.633)

95% credible intervals around each estimate are in brackets. Interval estimates which are distinct from 1 are highlighted in bold.

### Female Preference

We interpret variation in female preference using the mate acceptance data. The model with the highest support (98.6%) for this data indicates that genetic variation for both female preference (‘F’ term in Table 1) and male attractiveness (‘M’ term in Table 1) is important. The variance explained by both of these terms is high (Table 2). This is supported by the high heritability of female preference within each temperature (Table 3). The effect of temperature was included in the models to account for environmental variation, but there was no overall effect of temperature on preference (fixed effect estimate [with 95% credible interval] for 25°C: 1.45 [0.88–2.07]; and for 23°C: 1.39 [0.82–1.97]). However, there is genetic variation in plasticity of female preference across temperatures (i.e. a G×E component; Table 1 and Figure 1b). This G×E shows that the variation across temperatures in the proportion of males accepted as mates varied between female isolines. The variance explained by this G×E effect is fairly high (Table 2), consistent with the strong interaction shown in Figure 1b, and the weakened cross-environment genetic correlation and between-temperature heritability, which are both significantly lower than 1 (Table 3). In summary, there is genetic variation for both male attractiveness and female preference, and genetic variation for plasticity in female preference across temperatures.

The lack of F×M, M×t and F×M×t interactions in the best model for female preference (Table 1) shows that females of all genotypes and rearing temperatures tend to ‘agree’ on which male isolines are preferred, such that the ranked order of males does not change significantly between female isolines or environments (Figure 2b).

### Female Mate Choice for Male CHCs

The results of PCA on male CHC data gave us 4 PCs of CHC expression which together explain ca. 83% of the total variation in CHC profile. We used these vectors to reduce the dimensionality of the CHC data, whilst capturing a large proportion of the overall variation in CHC profile in order to describe female mate choice in terms of male phenotype. We do not examine CHC expression in detail, since we quantify cross-environment patterns of genetic variation in CHC profile in the same population of *D. simulans* isolines elsewhere [19], and very similar results are found from this data (analysis not shown).

Re-analysis of the set of models in Table 1 with the 4 PCs describing male CHC expression as covariates confirmed that using male isoline or male CHC data gives the same best model for choosiness (copulation latency) and preference (mate acceptance; results not shown). This was expected since we used an average CHC profile for each male isoline. The overall posterior estimates for *β* (with 95% credible interval) for each PC (Table 4) clearly show strong sexual selection on PC3 and PC4. Additionally, whilst PC2 does not significantly influence female preference, it does significantly influence female choosiness (Table 4).

**Table 4:** Overall estimates for *β*, the linear selection gradient, on each PC of CHC expression.

	*Choosiness*	*Preference*
PC1	−0.067 (−0.198–0.066)	0.083 (−0.284–0.449)
PC2	−**0.143 (**−**0.275–** −**0.013)**	0.015 (−0.372–0.395)
PC3	−**0.516 (**−**0.656–** −**0.377)**	**0.618 (0.214–1.022)**
PC4	**0.401 (0.310–0.493)**	−**0.460 (**−**0.717–** −**0.197)**

95% credible intervals around each estimate are in brackets. Interval estimates which are significantly different from 0 are highlighted in bold. Choosiness is inferred from copulation latency data and preference from mate acceptance data. Note that consistent sexual selection will have the opposite sign for choosiness and preference.

**Figure 3:**
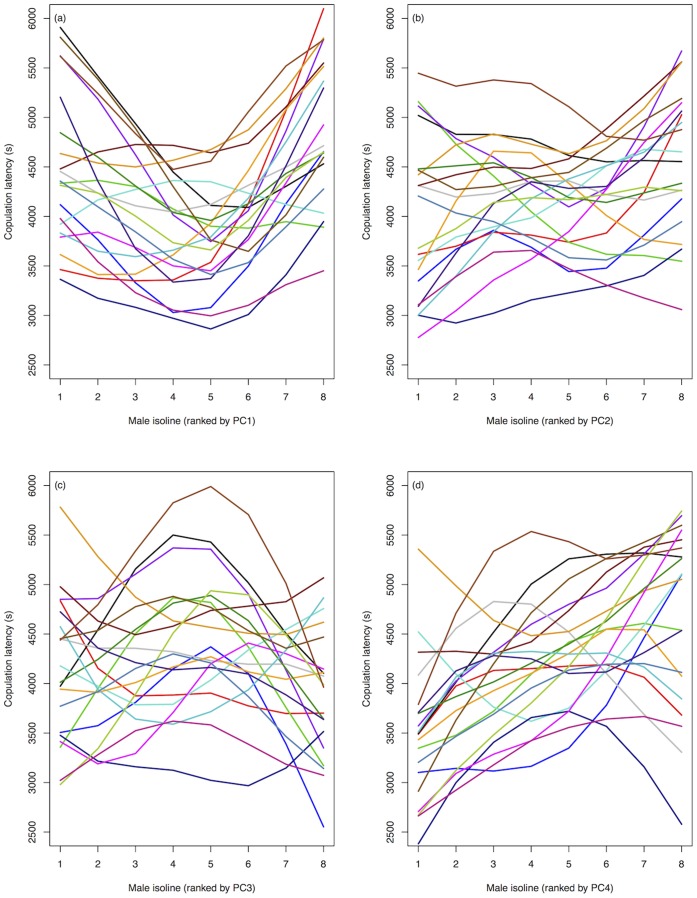
Genetic variation in female choosiness (copulation latency) as a function of male CHC profile (PCs 1–4, *(a)*–*(d)*). Male isolines (*N* = 8 isolines) are ranked on the x-axis according to mean PC score (left (low) to right (high) along axis). Each coloured line represents a female genotype (*N* = 20 isolines) pooled across temperatures.

**Figure 4:**
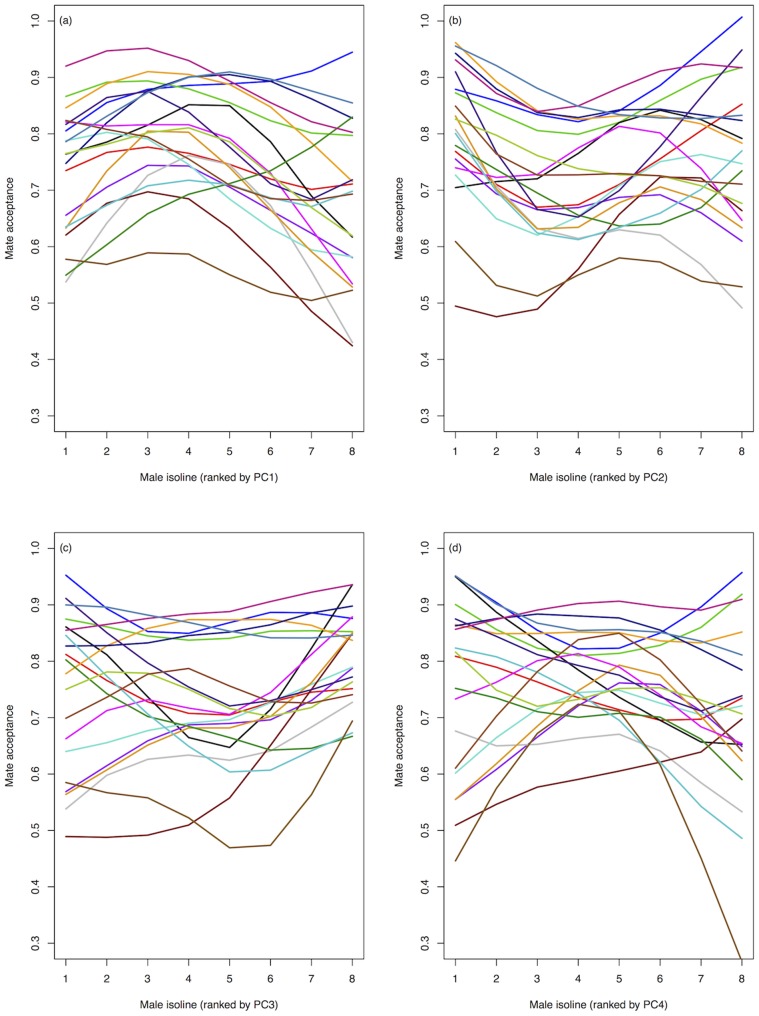
Genetic variation in female preference (mate acceptance) as a function of male CHC profile (PCs 1–4, *(a)*–*(d)*). Male isolines (*N* = 8 isolines) are ranked on the x-axis according to mean PC score (left (low) to right (high) along axis). Each coloured line represents a female genotype (*N* = 20 isolines) pooled across temperatures.

**Figure 5:**
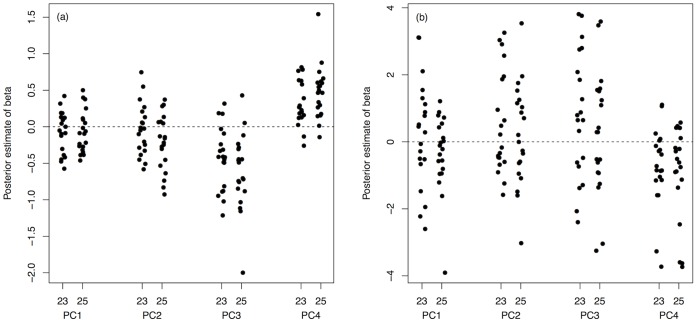
Posterior estimates of beta, *β*, the linear selection gradient, on each PC of male CHC expression for each female isoline×temperature combination using *(a)* female choosiness (copulation latency) and *(b)* female preference (mate acceptance) data. Each point represents a female isoline and the dashed line denotes *β* = 0 (i.e. no linear selection). Linear selection was significant overall on PC3 and PC4 for both choosiness and preference, and also on PC2 for choosiness (see text for details).

Our use of an average CHC profile per male isoline could have limited our ability to detect female G, E and G×E variation in mate choice for male CHCs, and so we examined mate choice on CHCs in more detail. Genetic variation in choosiness and preference (Figures 3 and 4, respectively) is illustrated as a function of each PC vector of CHC variation. There is potential for genetic variation in mate choice for CHCs, but further research will be necessary to clarify these results. Estimation of β for each female isoline and environment combination for each PC individually also shows some evidence for genetic variation in β (Figure 5), but again, further research will be needed.

### Genetic Covariance between Female Preference and Male Attractiveness

There was some statistical support for the model including a G×E across temperatures in male attractiveness score (DIC [posterior probability] = 1222.708 [0.707]), and the model including only G and E had lower support (DIC [posterior probability] = 1224.466 [0.293]). This suggests there is G×E in male attractiveness, however, the small change in DIC suggests the G×E effect is weak, and this is reflected in the cross-environment genetic correlation, which is high and only very marginally different from a correlation of 1 (0.977 [0.874–0.999]). Since male attractiveness score was calculated from female preference and male CHC expression data, this interaction could suggest the potential for the genetic correlation between female preference and male CHC attractiveness to vary across environments. However, genetic correlations both within (23°C: *r_g_* = 0.23 (−0.23–0.61), *P* = 0.165; 25°C: *r_g_* = 0.38 (−0.07–0.71), *P* = 0.063) and between temperatures (female 23°C, male 25°C: *r_g_* = 0.24 (−0.23–0.62), *P* = 0.156; female 25°C, male 23°C: *r_g_* = 0.10 (−0.36–0.52), *P* = 0.339), are positive but non-significant (although the correlation within 25°C is only marginally non-significant). We therefore find no evidence for genetic covariance between female preference and male attractiveness (as calculated from CHC profile) across any of the temperatures we studied.

## Discussion

Despite recent interest in the role of the environment and genotype-by-environment interactions in sexual selection, relatively little is known about the genetics of plasticity in female mate choice [12]. Here, we examine the genetics of two aspects of female mate choice, choosiness and preference, across two post-eclosion temperatures. We find evidence for genetic, environmental and G×E components of both choosiness and preference, making this one of a small number of studies to investigate the cross-environment genetics of mate choice behaviour [27], [28], [29]. However, the lack of variation in the ranked order of preferred male genotypes suggests that females from each isoline and environment generally agree on which males are most attractive, and so the outcome of mate choice is unlikely to differ across these temperatures (consistent with results from a recent study of *D. simulans* [20]).

The definitions used here for female choosiness and preference follow Jennions and Petrie [1] and are also consistent with Cotton et al [10]. The distinction between choosiness and preference can be useful, since female choosiness can vary (e.g. through changes in the costs or benefits of mate assessment) without necessarily altering overall preference [1]. Therefore, analysis of both choosiness and preference, as opposed to a univariate measure of mate choice, provides further insight into the evolution of female mate choice behaviours and the overall outcome of mate choice. Further, both choosiness and preference can vary without necessarily changing the ranked order of male genotypes or phenotypes [1]. Based on the G, E and G×E variation we identify, we consider the implications of our findings below.

### Female Choosiness

The best model for copulation latency identifies a genetic basis of both female choosiness and male attractiveness. Additionally, there was some evidence that females reared at the higher temperature are less choosy on average than females from the lower temperature, which was expected since it has been found previously that female *D. melanogaster* respond more quickly to males when kept at higher temperatures [43].

There was no significant genetic variation for plasticity in choosiness across temperatures (i.e. a female choosiness G×E was not included in the best statistical description of the data). However, the cross-environment genetic correlation and between-environment heritability provide evidence for substantial changes in genetic variation in choosiness between temperatures indicative of a G×E. The wide intervals around these estimates perhaps explain the lack of a significant statistical interaction, but the intervals overlap 0 and are distinct from 1 and so it is likely that there is some genetic variance in plasticity in female choosiness across temperatures. This G×E could mean that the evolution of this aspect of female mate choice will depend on the environment even across the narrow range of environmental variation assessed here, and note that this narrowness could also explain why the G×E term did not fall into the best-fit model. The results are therefore largely consistent with the only other studies we are aware of which test for G×E in female choosiness: Rodríguez and Greenfield [27] found a G×E for female responsiveness in *A. grisella* reared across different temperatures and Narraway et al [28] identified G×E for female choosiness in *D. melanogaster* dependent on temperature stress during development.

### Female Preference

From the mate acceptance data (preference), we find high genetic variance in female preference and male attractiveness. There is also substantial genetic variation in the effect of temperature, shown by a strong female preference G×E. The combination of high heritability and G×E variation means that there is considerable opportunity for the evolution of different female preferences, and variation in the strength of sexual selection, across environments. However, our results clearly demonstrate that the ranked order of preference for the different male genotypes does not differ across female genotypes or environments, nor with G×E. Therefore the ultimate outcome of mate choice does not vary across female genotypes or environments, and hence the same male genotypes are always preferred. Clearly, these results are based on a narrow range of environmental variation, and so the picture could potentially be very different if the environment varies on a different scale.

Interestingly, there was more evidence of a G×E for female preference than there was for choosiness. Possibly, the lack of overall temperature effect on preference means that there is little or no selection on female preference across temperatures, and thus genetic variation for plasticity across temperatures is maintained. Conversely, the stronger effect of temperature on choosiness could have eroded genetic variation for plasticity, creating a canalised response of choosiness across temperatures. This idea is supported by previous work that showed that female responsiveness varies predictably with temperature [32].

### Female Mate Choice for Male CHCs

The ability to detect significant G, E and G×E variation in mate choice for male CHCs could have been limited by the experimental design (by using an average male CHC profile per male isoline in the analysis). However, we were still able to quantify female mate choice for male CHC profiles across female genotypes and environments, and this reveals some interesting patterns underlying CHC attractiveness which potentially warrant further research. In particular, there is interesting genetic variation in female mate choice for aspects of male CHC profile. This is consistent with a study on *D. serrata* [29], where a genetic basis for female preference functions for male CHC profiles was identified, but there was no evidence of plasticity or G×E across a dietary manipulation.

Male CHCs function as sexual signals in a number of *Drosophila* species [29], [37] including *D. simulans* [41]. Consistent with these studies, we find evidence for significant sexual selection acting on 3 of the 4 vectors of CHC expression examined. Interestingly, PCs 3 and 4 are under selection using either component of mate choice (preference or choosiness), and therefore contribute to overall attractiveness of male CHC profile. On the other hand, PC2 only explains variation in female choosiness, perhaps indicating that CHC variation in this vector influences female responsiveness during courtship, rather than overall preference.

Despite the clear influence of male CHC profile on overall female mate choice, it is likely that there is sexually selected phenotypic variation in other traits which were not measured in this study, given that we did not assess other known elements of *Drosophila* courtship, such as song and dance [34]. Indeed, in previous work on this population of *D. simulans*, we found that despite complex patterns of G, E and G×E variation in male CHC profile [19], overall male attractiveness was strongly genetically determined and consistently heritable across a range of environments [20]. It would therefore appear that although CHCs influence female mate choice, the overall attractiveness of a given male probably correlates more strongly with male genotype than with a particular phenotypic trait, and that multiple sexual traits will affect a mating decision [34]. Indeed, it is also possible that some of our results could be attributed to female CHC profile. Our previous work found G, E and G×E variation in female CHC expression across these temperatures. Taken alongside research that has shown that female CHC profile might signal female receptivity to males [40], [44], it is possible that changes in female CHCs across the treatments in this study affected mating behaviour.

### Genetic Covariance between Female Preference and Male Attractiveness

Analysis of the genetic covariance between female preference and male attractiveness lends further support to the idea that multiple sexual traits contribute to the overall attractiveness of a given male. None of the genetic correlations measured between female preference and male CHC attractiveness were significant across any combination of temperatures, although they were consistently positive, as would be predicted under a Fisherian process. Similar results were also found in a study of the cross-environment genetic covariance between female preference and a male sexual signal in *A. grisella* [45]. At first glance, this is highly unexpected. In *D. simulans*, there is evidence for strong heritability of overall male attractiveness [9], [20] and female preference (this study), and additionally there is no evidence of any direct benefits of mate choice to females [30]. We therefore expect sexual selection to operate through a Fisherian runaway process, and a positive genetic correlation is expected to evolve between female preference and male attractiveness [6].

However, in the present study, male attractiveness (of males from the female isolines) was scored as a function of male CHC phenotype, and so the lack of covariance between female preference and male attractiveness could be an artefact of the complex multivariate nature of sexual signalling and preference. If females use multiple sexual signals to assess overall male attractiveness, then calculating male attractiveness scores from only the CHC data will overlook sexually selected variation in other male signals, thus resulting in the weakly positive genetic correlations we find between male attractiveness score and female preference. A more accurate method for scoring male attractiveness might therefore involve either measuring additional sexual traits, or the overall attractiveness of male genotypes.

A strong positive genetic correlation between preference and attractiveness is predicted to facilitate the runaway evolution of sexual traits [6], and so a weak correlation implies that although Fisherian sexual selection could operate, it is unlikely to result in accelerating trait evolution. In *D. simulans*, it seems likely that the strength of the genetic covariance between female preference and male CHC profile could be mediated by a combination of [1] indirect benefits of mate choice through heritable male attractiveness [9], [20], (2) multiple sexual signals contributing to overall variance in male attractiveness [20,34; this study], and (3) the balance between naturally and sexually selected optima in CHC profile. This balance is particularly relevant with respect to sexual selection across temperatures in *Drosophila*, given evidence that temperature-dependent natural selection will favour the production of CHCs that differ from those favoured by sexual selection [40], [41]. However, in our data there were no clear differences in the male-female genetic correlation across temperatures, and so it remains uncertain how important this factor is.

In conclusion, we find genetic, environmental and G×E variation in female choosiness and preference, but find no such variation in the ranked order of preferred males, such that the same male genotypes are likely to be favoured by sexual selection even across different environments and females. Therefore whilst the evolution of female mate choice behaviour could differ between these environments, the ultimate outcome of mate choice may be relatively consistent. Furthermore, the genetic covariance between female preference and male attractiveness, scored by CHC profile, is weak, and consistent with the idea that other male sexual signals contribute to overall attractiveness. This study highlights the importance of multivariate and cross-environment perspectives in order to gain a full understanding of sexual selection.
